# The Sitting-Height Index of Build, (Body Mass)/(Sitting Height)^3^, as an Improvement on the Body Mass Index for Children, Adolescents and Young Adults

**DOI:** 10.3390/children5020030

**Published:** 2018-02-22

**Authors:** Richard Burton

**Affiliations:** School of Life Sciences, College of Medical, Veterinary and Life Sciences, University of Glasgow, Glasgow G12 8QQ, UK; richard.burton@glasgow.ac.uk; Tel.: +44-(0)141-334-9738

**Keywords:** sitting height, leg length, sitting-height index of build, body mass index, BMI, Cormic index, adiposity rebound

## Abstract

The body mass index (BMI) is unsatisfactory in being affected by both relative leg length and height, and, for use with children and adolescents, therefore needs to be interpreted in relation to age. The sitting-height index of build (body mass)/(sitting height)^3^, is largely free of these disadvantages. Furthermore, because that index is independent of relative leg length, the latter can be treated as a separate indicator of nutritional history and health risks. Past studies on white children and adults have shown body mass to be approximately proportional to (sitting height)^3^. Moreover, multiple regression of (body mass)^1/3^ on sitting height and leg length, using year-by-year averages, has indicated that leg length is an insignificant predictor of body mass. The present study used data for individuals, namely 2–20 years old males and females, black as well as white. Regression analysis as above again showed leg length to be an insignificant predictor of body mass, but only above the age of about nine years. However, sitting height is still a stronger predictor of body mass than leg length at all ages. The advantages of the sitting-height index of build for use with young people are confirmed.

## 1. Introduction

Interpreting values of the body mass index (BMI) (body mass)/height^2^, for children and adolescents is not straightforward and typically involves re-expression in terms of centiles, z-scores, or percentages of the median for particular ages and reference populations [1]. This is because the BMI is influenced not only by body composition (of which it is taken as an indicator), but also by relative leg length (which increases markedly in the early years [2,3]. It also depends on scale at all ages due to height being squared rather than cubed [3,4] (see below). The sitting-height index of build (SHIB) (body mass)/(sitting height)^3^, has advantages over the BMI as an index of body mass status (i.e., in terms of “overweight”, “underweight” etc.), and this is especially so when applied to children and adolescents [3]. Thus, it is independent of both scale, and, to a large extent, leg length. Conveniently, leg length can therefore be used as a separate and independent indicator both of early nutrition and of health risks [2,5].

To approach the SHIB and BMI in terms of theory, it helps to consider first a hypothetical set of individuals of different sizes but identical density and bodily proportions. Their masses must vary in proportion to both (sitting height)^3^ and height^3^ [3,4], and the SHIB would be constant. The BMIs of these isometric individuals would inevitably increase with height (and therefore, in children, with age). For real people, body density varies little enough to be disregarded here despite its variation with body composition.

About a century ago (1916–1923), several authors related body mass to sitting height. Walker [6] did so because sitting height is comparable to the body lengths of quadrupeds. He found for ages 2 weeks to 20 years that year-by-year means of sitting height for both sexes increased approximately in proportion to (body mass)^1/3^. Bardeen [7] found means of the expression 100 × (body mass)/(sitting height)^3^ (g/cm^3^) to be nearly constant and uncorrelated with age in girls aged 6.5–17.5 years. (Related indices were also explored for adults at about the same time [8,9,10]). The exponents 1/3 and 3 were presumably chosen mainly on long-understood dimensional grounds [4], although that was not stated. Sometimes the primary interest in such indices has been in relation to respiratory function, for which upper body size would seem particularly relevant (e.g., [8]). For people of European descent, mean values of the SHIB have more recently been found to vary little between ages one and 25 years [3]. Data of Hamill et al. [11] for black and white males and females aged 12–17 years show only small and inconsistent changes in mean SHIB with age, the highest values for each category being only 4–7% higher than the lowest.

The evidence in the previous paragraph is consistent with growth that is nearly isometric, but childhood growth is far from isometric. Firstly, body mass may be affected by changes in adiposity. Secondly, the Cormic index, (sitting height)/height, has been found at first to fall steadily to a minimum at about 12–15 years and then tend to rise slightly before leveling off towards adulthood [3]. It is well established for adults that the BMI correlates with the Cormic index [12,13]. It has not become standard practice to adjust the BMI for variations in this ratio, but using the SHIB in place of the BMI would seem to be a way of achieving this end, since the SHIB is independent of leg length.

It may seem counterintuitive to relate body mass just to sitting height, as if the legs were without mass. Although leg length and sitting height are obviously correlated during growth, that is insufficient justification. Using four published sets of year-by-year data, Burton [3] regressed (mean body mass)^1/3^ on mean sitting height and mean leg length, finding that the regression coefficients for mean leg length were all non-significant. The proposed explanation was that leg mass varies almost in proportion to upper body mass and is largely independent of leg length because longer legs tend to be more slender [14].

The aims of the present study were to generalize the findings to other population samples, white and black, and to analyze relevant relationships among body mass, sitting height, leg length, and age using year-by-year sets of individual values, and not just sample means. The merits of the SHIB are confirmed.

## 2. Materials and Methods

The data are for non-Hispanic white and non-Hispanic black American children, adolescents, and young adults from the NHANES III Laboratory Data File (U.S. Department of Health and Human Services, 1988–1994) [15]. The measurement of sitting height is described in the NHANES procedures manual [16] and leg length (subischial) is defined as total height minus sitting height. The data are published with weightings and imputed values that can be used to improve the estimation of nationally representative statistics for the US, but that is not the objective here.

The relative importance of sitting height and leg length as determinants of body mass was explored by regression analysis. The following regression equation was applied to means for each age group by Burton [3]:

(body mass)^1/3^ = *a* × (sitting height) + *b* × (leg length) + *c* (1)

However, because the ratio *b*/*a* is of particular interest, the following rearrangement of Equation (1) was used in the present study, allowing for estimation of significance levels for *b*/*a*.

(body mass)^1/3^ = *a* × ((sitting height) + (*b*/*a*) × (leg length)) + *c* (2)

The cube roots of body mass were used for dimensional appropriateness [3,4] and to give near-linear relationships. The values of *a* and *c* are of little interest and are not recorded here. Also calculated were year-by-year mean values, with standard deviations (SDs), of sitting height, body mass, (sitting height)/height, SHIB, and BMI. Correlation coefficients for body mass and sitting height (*r*_BM.SH_) and for body mass and height (*r*_BM.height_) were compared in terms of their ratio, *r*_BM.SH_/*r*_BM.height_.

Statistical calculations were carried out using Excel 2007 (Microsoft Corporation, Redmond, WA, USA) and Datafit 8.0 (Oakdale Engineering, Oakdale, PA, USA). The units used were kg and m.

## 3. Results

Table 1, Table 2, Table 3 and Table 4 give sample sizes for the 76 data subsets, and the year-by-year means and SDs of relevant variables. All show a fall in the ratio (sitting height)/height to a minimum at about 10–13 years and then a slight rise towards adulthood. Minima at about 12–15 years have been found in other studies [3]. As is typical, the minima occurred earlier in the girls than the boys, this being at about the time of the adolescent growth spurt [17].

**Figure 1:**
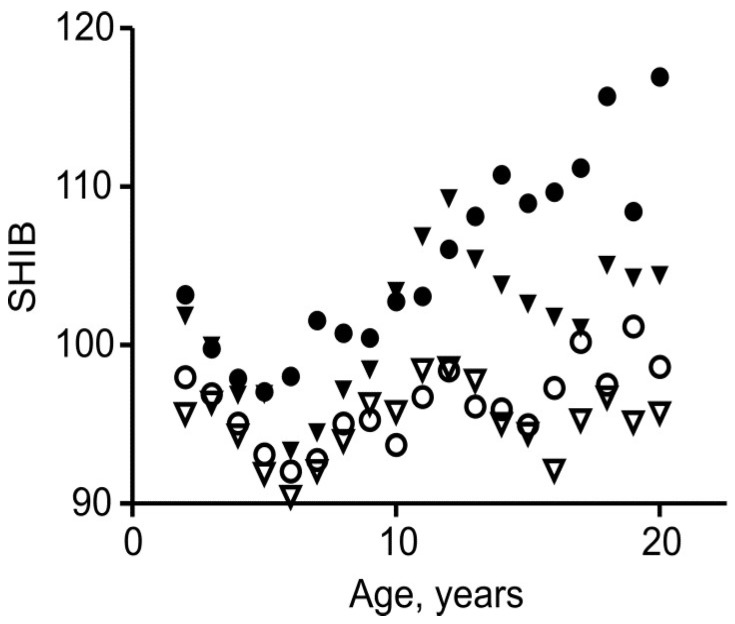
A plot of the sitting-height index of build, (body mass)/(sitting height)^3^ (SHIB), against age for white females (○), black females (●), white males (▽), and black males (▼).

Mean SHIB has previously been found to vary rather little with age, unlike mean BMI. Figure 1 shows the relationships between mean SHIB and age for the present data. For white males and females, the highest means were only 12% higher than the lowest over the full age range. The data for the black girls do show a marked upward trend, with mean SHIB increasing by 19% between the ages of 5 and 18 years, but this contrasts with an increase of 63% in mean BMI for these girls over the same age range. The corresponding increases in BMI for black boys, white girls, and white boys are, respectively, 52%, 43%, and 50%. Although the lesser dependence of SHIB on age is to be emphasized, significant trends in mean values are nevertheless to be expected due to variations in body composition (e.g., in fatness and muscularity) and in the proportions of the upper body. It is, of course, the dependence of both indices on body composition that makes them practically useful.

### 3.1. Analysis in Terms of Three Phases of Growth

The data are conveniently analyzed further in terms of three phases of growth. They are specified as age ranges, rather than being defined by developmental stages such as puberty.

#### 3.1.1. Phase III

**Figure 2:**
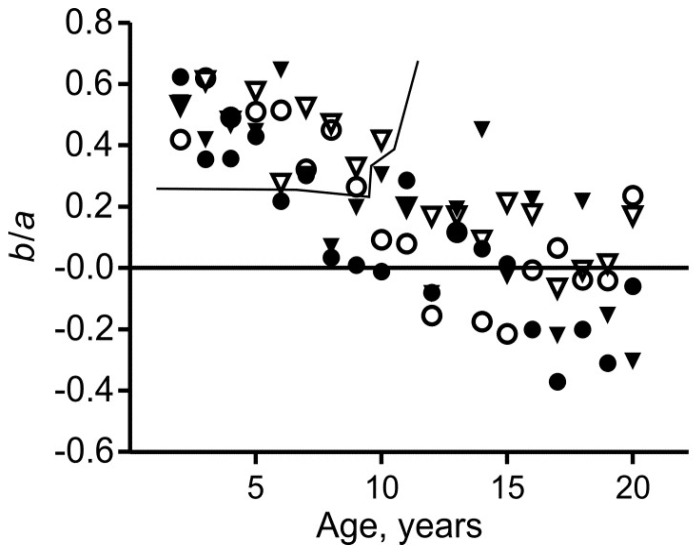
Values of the regression parameter *b*/*a* in Equation (2) plotted against age for white females (○), black females (●), white males (▽), and black males (▼). Values in the upper-left enclosed area differ significantly from zero (*p* < 0.01). The others do not (*p* > 0.05).

**Figure 3:**
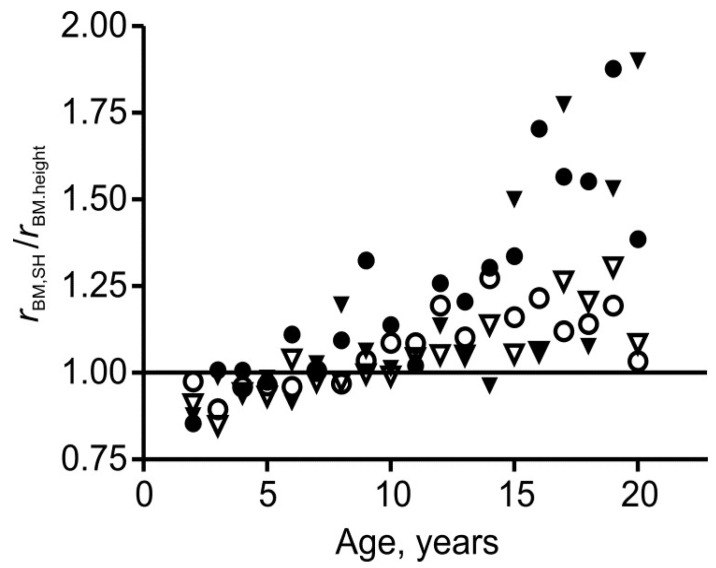
Comparison of sitting height and height as predictors of body mass at each age in terms of the ratio of correlation coefficients *r*_BM.SH_/*r*_BM.height_ for white females (○), black females (●), white males (▽), and black males (▼).

From the age of about 10 years upwards (phase III), *b*/*a* is near zero (Figure 2), the Cormic index is less age-dependent than in younger children, and, as noted above, mean SHIB is less age-dependent than the BMI. Body mass is generally more closely correlated with sitting height than with total height (Figure 3), indicating that percentage body fat should correlate more strongly with SHIB than with BMI. These features strongly favor use of the SHIB.

That *b*/*a* is near zero implies that longer legs tend to be more slender, as in adults [14]. Negative values may be entirely due to chance, but it could be relevant that Bogin and Varela-Silva [5] found sitting heights to correlate with gluteo-femoral fatness in black, white, and Mexican-American women and in black and Mexican-American men. The effect is small, but would slightly enhance the correlation between body mass and sitting height, and decrease the correlation between body mass and leg length.

Applying Equation (1), Burton [3] obtained values of *b*/*a* of −0.11 to +0.15. These were based on mean, rather than individual, values of the variables for the full age ranges, infant to young adult. Individual data for younger children (phase I) suggest a different picture.

#### 3.1.2. Phase I

In phase I, defined as ages 2–6 years, both the Cormic index, (sitting height)/height, and the Rohrer index, (body mass)/height^3^, fall most rapidly [2,3]. In phase I *b*/*a* was 0.22–0.65, with all but one value differing significantly from zero (Figure 2, *p* < 0.01). In all four groups, the SHIB fell consistently and significantly over time, and a feature seen both in Figure 1 and in Figure 2 of Burton [3] is a low mean SHIB at 5–6 years. Within this period, the mean values fell by 5.5–8.4%, with analysis of variance showing these falls to be highly significant (*p* ≤ 0.003). Nevertheless, the means for white subjects differed little from later values and the year-to-year differences were small, especially when compared with the standard deviations (Table 1, Table 2, Table 3, Table 4 and Figure 1). Moreover, the decreases within phase I were probably due to the steady decline in percentage fat content that has been found to occur from two to at least five years, similarly in girls and in boys [18]. In contrast to phase III, body mass was not generally more closely correlated with sitting height than with total height (Figure 3). Nevertheless, the general reasons, as already discussed, for favoring the SHIB over the BMI apply here and it is desirable that a single index be used for all ages.

The low mean SHIB at 5–6 years is suggestive of the well-known nadir in mean BMI at about that age that is followed by the so-called “adiposity rebound” [19], but it is not the same thing. The fall and rise, actually of BMI rather than adiposity, must be partly due to two opposing phenomena. One is the progressive increase in relative leg length then [2,3] that tends to lower the BMI. The other is the inherent tendency for BMI to increase with height. Variations in body composition could of course be relevant too. The data of Table 1, Table 2, Table 3 and Table 4 happen not to illustrate well the initial fall in mean BMI.

#### 3.1.3. Phase II

That the SHIB is appropriate for phases I and III implies the same for this intermediate phase also. The mean SHIB for all four groups rose by 2.6–6.5% between the ages of 6 and 9 years (Figure 1); In accordance with their inherent scale-dependence, the mean BMIs rose proportionately more—1.8–4.0 times those percentage rises in mean SHIB.

### 3.2. Comparing Black and White, Females and Males

The aim of this study was not that of comparing these four groups, but of checking the generality of conclusions about the SHIB. Nevertheless, Figure 1 does show differences in mean SHIB. Thus, it was higher in the black males and females, as may also be shown for the data of Hamill et al. [11] for ages 12–17 years. Mean SHIB also tended to be higher in the older females than in the corresponding males, a difference that is probably related largely to fat content. As noted above, boys and girls tend to differ in the timing of changes in relative leg length.

The variations in mean SHIB with age shown in Figure 1 match to some extent the trends in the percentage of overweight individuals, as assessed by BMI centiles in 1999–2000 [20]. In the non-Hispanic whites there was little change with age, while upward trends were evident in the non-Hispanic black boys and girls. For ages 6–19 years, percentages of overweight were higher in the non-Hispanic blacks than the non-Hispanic whites, especially for ages 12–19 years. This is evidence that the trends and differences in SHIB relate to adiposity, as expected. A useful new approach to this issue would be to relate values of (fat-free mass)/(sitting height)^3^ to age and sex.

## 4. Discussion

The present study, using individual as well as just mean data, as previously, confirms that the SHIB is better in various respects than the BMI as an index of body mass status (“overweight”, “underweight” etc.) from the age of two years to adulthood. However, because the BMI is so well entrenched in clinical practice and the public mind, it may be appropriate to record both together in research reports. This would allow further comparison of their merits.

Exact inter-conversion between the two indices would not of course be needed if both height and sitting height were measured for each individual. However, BMI “cut-off” values defining “overweight”, “obesity”, etc. (which are to some extent arbitrary), are easily converted to SHIB values. The ratio SHIB/BMI equals height^2^/SH^3^ and the mean of this ratio supplies the necessary conversion factor for a given age. For example, based just on the 20-year-old white men of Table 1, the conversion factor would be 4.0. If the lower cut-off BMI value defining “overweight” for white adults is taken as 25 kg/m^2^ [21], the equivalent value for SHIB is 100 kg/m^3^.

Several issues remain to be explored. The near-zero values of *b*/*a* in adults have been explained as a consequence of the tendency of long legs to be more slender [14]. That is probably true for younger people also, but that has not been tested. Next, it is to be expected that percentage body fat would correlate more closely with SHIB than with BMI or BMI centiles, but this too has yet to be demonstrated and quantified. It is suggested above that certain variations in mean SHIB reflect fat content; it would be useful to know how far that is true of these and other variations. Given data on fatness, one could also explore the presumably lower variability of the ratio (fat-free mass)/(sitting height)^3^. As for differences among ethnic groups, the means of BMI and Cormic index [22] in adults vary considerably and are significantly correlated [23]. This implies that the means of SHIB (which are independent of Cormic index) vary less than those of BMI. Thus, an important question is whether relationships between percentage fat content and SHIB are more similar for different populations of children and adolescents than between percentage fat content and BMI. It would be especially interesting to study populations with widely different mean Cormic indices, such as Chinese and East Africans [23]. Finally, the BMI serves as a predictor not only of body mass status and adiposity, but also of health risks and mortality. If the risks relate to body mass status, as is generally assumed, then the SHIB should be the better risk predictor. Quantitative data on the various relationships would be invaluable.

Although versions of the SHIB were formulated a century ago [6,7,8,9,10], they have generally been ignored. Their more recent neglect could be due to the obvious contribution of the legs to body mass and the apparent tendency of the BMI to be seen as a physically meaningful property of the body, like density, rather than as a statistical construct that is based mainly on adult data [24].

## Figures and Tables

**Table 1:** Sample sizes and means (with standard deviations in brackets) of sitting height (cm), body mass (kg), ratio of sitting height to standing height, sitting-height index of build (SHIB, kg/m^3^) and body mass index (BMI, kg/m^2^) of white males. The data are from the NHANES III laboratory data file [15].

Age, Years	Number	Sitting Height	Body Mass	(Sitting Height)/Height	SHIB	BMI
2	369	52.3 (2.2)	13.7 (1.7)	0.575 (0.017)	95.7 (9.6)	16.5 (1.3)
3	293	54.9 (2.4)	15.9 (2.3)	0.555 (0.019)	96.4 (11.9)	16.2 (1.6)
4	330	57.7 (2.7)	18.2 (3.1)	0.546 (0.016)	94.3 (11.5)	16.2 (1.9)
5	296	60.5 (2.8)	20.4 (3.4)	0.540 (0.014)	91.9 (12.9)	16.1 (2.1)
6	150	63.4 (3.3)	23.1 (4.6)	0.535 (0.018)	90.4 (11.7)	16.4 (2.4)
7	159	66.3 (3.7)	27.1 (6.6)	0.528 (0.014)	92.1 (15.2)	17.0 (3.0)
8	164	68.5 (3.4)	30.5 (7.8)	0.523 (0.014)	94.0 (16.8)	17.6 (3.4)
9	161	71.3 (3.1)	35.3 (9.2)	0.520 (0.013)	96.3 (17.3)	18.5 (3.6)
10	164	73.3 (3.7)	38.1 (9.7)	0.519 (0.017)	95.8 (16.5)	18.9 (3.6)
11	160	75.1 (4.3)	42.4 (12.5)	0.514 (0.012)	98.4 (18.1)	19.6 (4.3)
12	112	78.6 (4.4)	48.3 (12.7)	0.512 (0.012)	98.6 (17.7)	20.3 (4.1)
13	116	82.7 (4.8)	55.7 (14.7)	0.513 (0.011)	97.8 (20.0)	21.3 (4.6)
14	96	86.0 (4.9)	60.2 (14.4)	0.514 (0.014)	95.0 (20.0)	21.5 (4.6)
15	93	88.2 (4.7)	65.1 (15.4)	0.518 (0.014)	94.4 (17.3)	22.3 (4.3)
16	110	90.4 (4.0)	68.2 (16.5)	0.520 (0.012)	92.1 (17.2)	22.5 (4.5)
17	121	90.5 (4.0)	70.8 (13.9)	0.523 (0.013)	95.3 (14.9)	23.6 (4.0)
18	105	91.1 (3.6)	73.2 (15.4)	0.524 (0.014)	96.7 (18.3)	24.2 (4.7)
19	79	91.2 (3.5)	72.1 (12.8)	0.525 (0.013)	95.1 (15.6)	23.9 (4.0)
20	105	90.4 (3.8)	70.7 (13.1)	0.526 (0.012)	95.7 (15.6)	23.9 (3.9)

**Table 2:** Sample sizes and means (with standard deviations in brackets) of sitting height (cm), body mass (kg), ratio of sitting height to standing height, sitting-height index of build (SHIB, kg/m^3^) and body mass index (BMI, kg/m^2^) of white females. The data are from the NHANES III laboratory data file [15].

Age, Years	Number	Sitting Height	Body Mass	(Sitting Height)/Height	SHIB	BMI
2	392	51.5 (2.4)	13.4 (2.0)	0.573 (0.016)	98.0 (10.5)	16.5 (1.7)
3	348	54.3 (2.8)	15.5 (2.2)	0.554 (0.019)	96.9 (12.3)	16.1 (1.5)
4	327	57.1 (2.6)	17.7 (3.1)	0.545 (0.015)	95.1 (13.1)	16.1 (2.1)
5	327	60.1 (3.1)	20.3 (3.9)	0.538 (0.015)	93.1 (12.3)	16.1 (2.2)
6	165	62.3 (3.3)	22.5 (5.1)	0.529 (0.014)	92.0 (14.3)	16.0 (2.6)
7	168	65.2 (3.3)	26.1 (6.7)	0.528 (0.013)	92.8 (15.4)	16.9 (3.1)
8	151	68.0 (3.6)	30.2 (8.5)	0.523 (0.013)	95.1 (18.7)	17.7 (3.7)
9	167	70.6 (3.7)	34.1 (9.3)	0.521 (0.014)	95.3 (16.0)	18.3 (3.7)
10	155	73.5 (3.9)	37.5 (8.8)	0.518 (0.012)	93.7 (15.2)	18.5 (3.4)
11	174	77.5 (4.2)	45.6 (11.0)	0.516 (0.012)	96.7 (15.7)	20.0 (3.8)
12	112	80.5 (4.5)	51.7 (12.8)	0.520 (0.013)	98.4 (19.8)	21.5 (4.8)
13	132	82.8 (4.1)	54.9 (12.9)	0.526 (0.014)	96.1 (18.6)	22.0 (4.4)
14	134	84.2 (3.7)	57.4 (11.6)	0.527 (0.012)	96.0 (16.5)	22.5 (4.2)
15	112	85.6 (4.4)	59.2 (11.4)	0.530 (0.019)	95.0 (18.4)	22.7 (4.3)
16	119	85.1 (3.1)	60.2 (14.9)	0.530 (0.012)	97.3 (21.3)	23.3 (5.3)
17	119	85.9 (3.3)	63.3 (14.7)	0.530 (0.012)	100.2 (23.0)	24.2 (5.5)
18	106	84.9 (3.9)	59.6 (12.4)	0.528 (0.012)	97.5 (19.2)	23.0 (4.5)
19	120	85.4 (3.7)	63.1 (15.1)	0.531 (0.012)	101.2 (22.9)	24.3 (5.6)
20	103	85.6 (3.5)	62.0 (13.6)	0.531 (0.012)	98.6 (18.3)	23.8 (4.5)

**Table 3:** Sample sizes and means (with standard deviations in brackets) of sitting height (cm), body mass (kg), ratio of sitting height to standing height, sitting-height index of build (SHIB, kg/m^3^) and body mass index (BMI, kg/m^2^) of black males. The data are from the NHANES III laboratory data file [15].

Age, Years	Number	Sitting Height	Body Mass	(Sitting Height)/Height	SHIB	BMI
2	195	51.3 (2.5)	13.7 (1.6)	0.562 (0.022)	101.9 (18.4)	16.4 (1.5)
3	166	54.4 (2.7)	16.0 (2.1)	0.543 (0.016)	99.9 (10.0)	16.0 (1.3)
4	178	57.5 (2.6)	18.4 (2.6)	0.534 (0.016)	96.8 (10.5)	15.8 (1.5)
5	162	60.2 (3.0)	21.2 (3.9)	0.525 (0.014)	96.9 (11.1)	16.0 (2.0)
6	85	62.9 (3.2)	23.3 (4.7)	0.522 (0.014)	93.3 (12.9)	15.9 (2.3)
7	94	65.8 (3.3)	27.2 (6.1)	0.516 (0.013)	94.5 (12.8)	16.6 (2.7)
8	86	67.9 (3.0)	30.6 (6.7)	0.512 (0.014)	97.1 (15.9)	17.4 (3.3)
9	103	70.3 (3.2)	34.5 (9.2)	0.505 (0.012)	98.4 (18.9)	17.7 (3.7)
10	103	71.7 (4.1)	38.9 (11.5)	0.500 (0.013)	103.4 (18.5)	18.6 (3.9)
11	95	74.9 (4.3)	45.6 (13.5)	0.498 (0.010)	106.8 (19.8)	19.9 (4.3)
12	78	76.9 (4.3)	50.9 (17.2)	0.497 (0.012)	109.2 (23.3)	20.9 (5.4)
13	56	81.1 (5.1)	57.1 (18.0)	0.497 (0.011)	105.4 (23.2)	21.2 (5.3)
14	72	85.0 (4.5)	64.0 (19.0)	0.501 (0.013)	103.8 (26.7)	22.1 (5.9)
15	69	86.6 (3.5)	67.1 (17.6)	0.502 (0.015)	102.6 (23.2)	22.4 (5.7)
16	71	87.3 (3.8)	68.4 (15.4)	0.504 (0.015)	101.8 (17.5)	22.6 (4.3)
17	63	88.4 (3.7)	69.9 (14.9)	0.509 (0.014)	101.1 (18.8)	23.2 (5.0)
18	59	90.0 (3.6)	77.4 (19.7)	0.507 (0.012)	105.0 (20.5)	24.4 (5.2)
19	65	89.1 (3.7)	74.1 (16.4)	0.507 (0.013)	104.2 (19.1)	24.0 (5.1)
20	38	88.2 (3.8)	71.9 (17.4)	0.509 (0.013)	104.4 (21.0)	23.9 (5.7)

**Table 4:** Sample sizes and means (with standard deviations in brackets) of sitting height (cm), body mass (kg), ratio of sitting height to standing height, sitting-height index of build (SHIB, kg/m^3^) and body mass index (BMI, kg/m^2^) of black females. The data are from the NHANES III laboratory data file [15].

Age, Years	Numbers	Sitting Height	Body Mass	(Sitting Height)/Height	SHIB	BMI
2	145	50.3 (2.3)	13.1 (1.8)	0.556 (0.019)	103.2 (11.7)	16.0 (1.4)
3	198	53.8 (2.5)	15.6 (2.7)	0.542 (0.015)	99.8 (10.8)	15.7 (1.8)
4	160	56.9 (2.7)	18.1 (2.8)	0.534 (0.014)	97.9 (10.7)	15.8 (1.7)
5	175	59.8 (3.0)	20.8 (3.6)	0.524 (0.016)	97.1 (13.0)	15.9 (2.1)
6	92	63.0 (3.6)	24.8 (6.9)	0.520 (0.020)	98.0 (17.3)	16.7 (3.5)
7	88	65.2 (3.3)	28.5 (7.8)	0.513 (0.012)	101.6 (19.9)	17.5 (3.7)
8	81	68.3 (3.9)	32.5 (8.4)	0.508 (0.012)	100.8 (16.7)	17.8 (3.5)
9	93	70.6 (3.6)	35.9 (9.3)	0.505 (0.021)	100.5 (16.0)	18.2 (4.0)
10	92	73.5 (4.0)	41.3 (11.1)	0.504 (0.012)	102.8 (19.1)	19.2 (4.3)
11	91	77.1 (4.5)	47.9 (12.7)	0.505 (0.014)	103.1 (20.2)	20.3 (4.3)
12	89	80.1 (4.0)	54.9 (14.8)	0.506 (0.014)	106.0 (22.3)	21.8 (5.2)
13	82	82.2 (3.3)	60.4 (14.7)	0.509 (0.016)	108.1 (21.7)	23.1 (5.1)
14	64	82.4 (3.3)	62.6 (18.9)	0.508 (0.016)	110.8 (27.6)	23.7 (6.5)
15	61	83.7 (3.5)	64.3 (14.9)	0.512 (0.015)	109.0 (21.4)	24.0 (5.2)
16	88	83.8 (3.5)	64.8 (16.1)	0.513 (0.014)	109.7 (23.9)	24.3 (5.9)
17	70	83.8 (4.0)	65.3 (17.4)	0.515 (0.012)	111.2 (28.2)	24.7 (6.4)
18	71	84.0 (3.6)	69.4 (22.6)	0.515 (0.013)	115.7 (31.6)	25.9 (8.0)
19	59	85.3 (3.2)	67.3 (16.5)	0.517 (0.011)	108.4 (24.2)	24.7 (6.1)
20	66	84.0 (3.3)	70.0 (18.9)	0.514 (0.014)	116.9 (25.4)	26.0 (6.4)
